# Periodontal risk assessment in a teaching hospital population in Saudi Arabia’s Eastern Province

**DOI:** 10.1016/j.sdentj.2021.09.014

**Published:** 2021-09-13

**Authors:** Marwa Madi, Afsheen Tabasum, Ahmed Elakel, Deamah Aleisa, Nabras Alrayes, Hend Alshammary, Intisar Ahmad Siddiqui, Khalid Almas

**Affiliations:** a Department of Preventive Dental Sciences, College of Dentistry, Imam Abdulrahman Bin Faisal University, Dammam, Kingdom of Saudi Arabia; b College of Dentistry, Imam Abdulrahman Bin Faisal University, Dammam, Kingdom of Saudi Arabia; c Department of Dental Education, College of Dentistry, Imam Abdulrahman Bin Faisal University, Dammam, Kingdom of Saudi Arabia

**Keywords:** Periodontal disease, Periodontal Risk Assessment, Teaching hospital, Diabetes, Smoking, Systemic condition, Saudi Arabia

## Abstract

**Objective:**

With this cross-sectional study, we aimed to evaluate factors associated with moderate and high risk of periodontal disease (PD) progression in the Saudi population.

**Methods:**

We reviewed 281 patients’ clinical charts from predoctoral periodontal clinics at the dental teaching hospital in the College of Dentistry (COD) at Imam Abdulrahman Bin Faisal University (IAU) in Dammam, Saudi Arabia. After obtaining ethical approval, we determined the Periodontal Risk Assessment (PRA) of the included patients based on the modified criteria developed by Lang and Tonetti (2003). We used logistic regression on stratified data and divided the results into two categories (low-moderate and high risk) to assess the effect modifier for potential risk factors. We used SPSS version 22 for data analysis, and considered a P-value ≤ 0.05 to be statistically significant.

**Results:**

Out of the 281 patients, 104 (37.0%) were male and 177 (63.0%) were female, with a mean age of 39.9 ± 14.0 years; 78.1% were Saudi nationals, 77% were married, and 44.6% were in the age group of 30 to 49. The PRA revealed 86 (30.5%) to represent high risk, 108 (38.3%) denoted moderate risk, and 88 (31.2%) signaled low risk for periodontitis. Logistic regression analysis showed that males were three times more likely to have high PRA (OR = 3.24) and to be married (OR = 2.77), as well as to be active smokers (OR = 8.87). The highest predictive factors of high PRA were 8 or more pockets ≥ 5 mm (OR = 29.0), those with active diabetes mellitus (DM; OR = 10.2), and those with 8 or more missing teeth (OR = 9.15).

**Conclusion:**

Saudi males who are married and have residual periodontal pockets, are actively diabetic, and with missing teeth are at high risk of PD. Further research is needed with a larger sample size comparing the general population with and without PD.

## 1 Introduction

Periodontal disease (PD) is a pathology of humans’ oral cavity that has been identified globally (Bostanci and Belibasakis, 2018). Periodontitis and gingivitis constitute PD, are accountable for the destruction of the supporting tissues of the tooth apparatus and are the major causes of tooth loss among adults (Kinane et al., 2017).

PD is the 11th most prevalent condition worldwide (Roberts et al., 2019). A review of 75 studies indicated that at the age of 40, the prevalence of severe periodontitis peaks and remains stable in older ages; thus, the older population exhibits an increased burden of disease (Kassebaum et al., 2014, Tonetti et al., 2017).

There is a lack of recent data regarding the prevalence of PD among the Saudi population (Alshammari and Wahi, 2019). Few studies suggest that the rates of oral health conditions are high; however, these findings are very general, and more studies must be performed to assess the prevalence of the PD in particular (Claffey et al., 1990, Idrees et al., 2014).

A clinical diagnosis must be made after reaching the treatment goals of active periodontal therapy, and the diagnosis must be based on the patient’s health status after periodontal health is restored. Hence, establishing new baseline parameters and assessing the risk level for progression of the disease in every individual patient will enable the clinician to determine the extent and frequency of supportive periodontal therapy to maintain periodontal attachment levels during post-active therapy (Bouchard et al., 2016).

Several risk factors are associated with the development of PD. It is well established that plaque biofilm and poor oral hygiene are the major determinants of the incidence of PD; thus, any other factors that impair the proper removal of biofilm are also considered risk factors (Lang and Tonetti, 2003). Such factors include anatomical anomalies, tartar, and restoration-related mishaps (Knight et al., 2016). In addition, smoking, diabetes, and low socioeconomic status are genuine risk factors for PD. All these risk factors are modifiable, but there are non-modifiable factors such as age, gender, and ethnicity (Almerich-Silla et al., 2017, Leite et al., 2018, Nascimento et al., 2018).

After completing active periodontal treatment (APT), most periodontitis patients are still at risk of further disease progression or even relapse (Ferraiolo, 2016). We defined disease progression using several criteria, such as attachment loss of ≥ 1.3 – 2 mm and other uses ≥ 3 mm (Tonetti and Claffey, 2005, Graetz et al., 2017).

A sound measure of the result of periodontal progression is tooth loss. As such, many publications assess risk factors for tooth loss due to progressive attachment loss (Müller et al., 2013, Pretzl et al., 2018).

Various periodontal risk assessment methods have been employed to determine patients’ probability of suffering from disease progression (Dhulipalla et al., 2015, Trombelli et al., 2017). The two most used tools (Sai Sujai et al., 2015) are the periodontal risk calculator (PRC) developed by Page et al. (Page et al., 2002), and the Periodontal Risk Assessment (PRA) created by Lang and Tonetti (2003).

The PRC is mainly based on mathematical calculations that assign relative weights to 11 factors and enable the stratification of results into 5 categories (1 = very low risk to 5 = very high risk) (Page et al., 2002). The PRA, on the other hand, uses 6 factors related to the progression of periodontitis. The outcome of the PRA is the individual risk stratification, divided into 3 categories (low, moderate, and high risk) (Lang and Tonetti, 2003). Knowledge of the risk of disease progression may be practically used to assign supportive periodontal therapy (SPT) intervals or to control modifiable risk factors (Sai Sujai et al., 2015).

Thus, with this cross-sectional study, we aimed to evaluate factors associated with moderate and high risk of PD progression in the Saudi population.

## 2 Methodology

We conducted this cross-sectional using records of patients who visited the periodontology clinics at the dental teaching hospital of Imam Abdulrahman Bin Faisal University’s (IAU) College of Dentistry (COD) for periodontal treatment from September 2018 to December 2019. IAU’s COD approved the study’s protocol, and we obtained approval from the research ethics committee (#2018024), as well as written informed consent from all participants. We performed the study in accordance with the Strengthening the Reporting of Observational Studies in Epidemiology (STROBE) guidelines.

### 2.1 Eligibility criteria

The inclusion criteria for the patients’ records were the presence of a complete periodontal chart and past medical history.

### 2.2 Exclusion criteria

The exclusion criteria were the reporting of malignancy, pregnancy, breastfeeding, taking medications that affect bone turnover, an incomplete periodontal chart, periodontal surgery within the past year, the absence of radiographs with good quality, and antibiotic use within 3 months prior to the dental visit.

### 2.3 Clinical parameters

We gathered demographic data on traits such as gender, age, nationality, and occupation. Information on dental health behavior such as dental visits, brushing and flossing habits were collected. We also collected details about each patient’s past medical history—including diabetes mellitus (DM), hypertension (HTN), cardiovascular disease (CVD), and medications—from his/her medical records. We also noted smoking habits; we defined current smokers as subjects who smoked or had stopped smoking less than 12 months before enrolling in the study, while former smokers were subjects who had quit smoking more than 12 months prior.

The collected periodontal parameters were periodontal pocket depth (PPD) and clinical attachment loss (CAL), measured using the UNC-15 on six sites per tooth, excluding third molars. We measured bleeding on probing (BOP) (Lang et al., 1990); then, we calculated the percentage of BOP. We also collected information on the number of missing teeth due to PD and the amount of bone loss.

PD severity based on Caton et al.’s classification (2018). We defined periodontitis as having>2 detectable interproximal CAL. Progression occurs in the following stages:

• Stage I: initial periodontitis (the greatest interproximal CAL = 1–2 mm, and RBL < 15%)
• Stage II: moderate periodontitis (CAL = 3–4 mm, and RBL = 15–33%)
• Stage III: severe periodontitis (CAL ≥ 5 mm, and RBL ≥ 30%)
• Stage IV: severe periodontitis with the potential for edentulism (CAL ≥ 5 mm, and RBL ≥ 50%)

We then computed the PRA for each patient based on the data gathered for the following five parameters, and performed calculations using a tabular form:

(1) Percentage of sites with BOP (<10%, 10%–25%, and >25%)
(2) Number of residual pockets ≥ 5 mm
(3) Number of lost teeth, except for third molars (28 teeth) (Lang and Tonetti, 2003)
(4) Cigarette consumption
(5) Presence or absence of diabetes

Out of 400 records, we included the files of 281 patients. We excluded other files due to an incomplete past medical history or periodontal chart, or the cause of missing teeth was not stated. Out of these, 104 patients were male and 177 were female. The mean age ranged from 16 to 82 years old. The mean age was 39.9 ± 14.0 years.

### 2.4 Statistical analysis

We analyzed the data using SPSS version 20.0(USA). We stratified numeric variables (including age) and PRA parameters per reference value criteria. Using standard cutoff values, we classified categorical variables (e.g., gender, nationality, marital status, occupation, medical history, DM, smoking), stratified variables (e.g.,age), and PRA parameters, and presented them as frequencies and percentages. We applied a chi-square test to compare all these variables between high, moderate, and low PRA categories. We used logistic regression analysis to evaluate the predictive factors of high PRA based on the severity of PRA as a binary dependent variable (i.e.,high and moderate PRA) and a panel of 12 independent covariates. We considered a P-value ≤ 0.05 to be statistically significant. Further, we adjusted aspects of lifestyle including diet, exercise habits, and socioeconomic factors as confounders.

## 3 Results

We evaluated a total of 281 patients for PRA; out of them, 104 (37.0%) were male and 177 (63.0%) were female, with a male-to-female ratio of 1:1.7. The mean age was 39.9 ± 14.0 (ranging from 16 to 82) years, and 44.6% of patients were 30 to 49 years old. Most of the patients were Saudi nationals (78.1%), and more were married (77%) than single. The PRA revealed that 85 (30.5%) were at high risk, 108 (38.3%) were at moderate risk, and 88 (31.2%) were at low risk for periodontitis.

All the demographic traits show significant differences of proportions between the high versus moderate and low categories of the severity of PRA. These data indicate a significant relationship between high PRA and being male, a Saudi national, over age 40, married, and employed (p ≤ 0.001). There was also a significant relationship of high PRA with a past medical history of DM, HTN, and CHD (Table 1).

**Table 1:** The relationship of PRA with demographic traits and past medical history.

Patient characteristics			Periodontal risk assessment (PRA)			P-value
		Total	High (n = 85)	Moderate (n = 108)	Low (n = 88)	
Gender	Male	104 (37.0)	48 (56.5)*	33 (30.6)	23 (26.1)	≤0.001
	Female	177 (63.0)	37 (43.5)	75 (69.4)	65 (73.9)	
Nationality	Saudi	218 (78.1)	67 (78.8)*	79 (73.8)	72 (82.8)	≤0.001
	Non-Saudi	61 (21.9)	18 (21.2)	28 (26.2)	15 (17.2)	
Age group	≤40 years	139 (49.5)	24 (28.2)	52 (48.1)	63 (71.6)	≤0.001
	>40 years	142 (50.5)	61 (71.8)*	56 (51.9)	25 (28.4)	
Marital status	Single	62 (23.0)	10 (12.2)	20 (19.4)	32 (38.1)	≤0.001
	Married	207 (77.0)	72 (87.8)*	83 (80.6)	52 (61.9)	
Occupation	Employed	89 (31.7)	40 (47.1)*	34 (31.5)	15 (17.0)	0.010
	Household dependent	192 (68.3)	45 (52.9)	74 (68.5)	73 (83.0)	
Medical history	DM	35 (12.5)	25 (29.4)*	10 (9.3)	0 (0)	≤0.001
	HTN	33 (11.7)	18 (21.2)*	9 (8.3)	6 (6.8)	
	CHD	6 (2.1)	5 (5.9)*	0 (0)	1 (1.1)	
	Other	39 (13.9)	7 (8.2)	10 (9.3)	22 (25.0)	
	None	168 (59.8)	30 (35.3)	79 (73.1)	59 (67.0)	

* Shows a significantly higher proportion at the 5% level of significance (based on the chi-square test). Values given in parentheses are percentages. DM = diabetes mellitus, HTN = hypertension, CHD = coronary heart disease.

All PRA risk factors exhibited significant proportions between the high versus moderate and low categories of the severity of PRA (p ≤ 0.001). These data provide sufficient evidence of the link between high PRA with active DM, smoking, a BOP > 25%, more than 8 pockets > 5 mm, and more than 8 missing teeth (Table 2).

**Table 2:** The relationship of PRA with various periodontal risk factors.

Factors			Periodontal risk assessment (PRA)			
		Total	High (n = 85)	Moderate (n = 108)	Low (n = 88)	P-value
Diabetes	Yes	36 (12.8)	27 (31.4)*	9 (8.3)	0 (0)	≤0.001
						
	No	246 (87.2)	59 (68.6)	99 (91.7)	88 (100)	
						
Smoking	Yes	51 (18.1)	36 (42.4)*	13 (12.0)	2 (2.3)	≤0.001
						
	No	230 (81.9)	49 (57.6)	95 (88.0)	86 (97.7)	
BOP %	> 25% (High)	109 (38.9)	57 (67.1)*	40 (37.0)	12 (13.8)	≤0.001
	10%–25% (Moderate)	115 (41.1)	22 (25.9)	47 (43.5)	46 (52.9)	
	< 10% (Low)	56 (20.0)	6 (7.1)	21 (19.4)	29 (33.3)	
No. of pockets ≥ 5 mm	> 8 (High)	52 (18.7)	45 (52.3)*	7 (6.6)	0 (0)	≤0.001
	5 – 8 (Moderate)	52 (18.7)	15 (17.4)	35 (33.0)	2 (2.3)	
	≤ 4 (Low)	174 (62.6)	26 (30.2)	64 (60.4)	84 (97.7)	
Missing teeth	> 8 (High)	32 (11.5)	24 (28.2)*	8 (7.5)	0 (0)	0.002
	5 – 8 (Moderate)	47 (16.8)	15 (17.6)	25 (23.6)	7 (8.0)	
	≤ 4 (Low)	200 (71.7)	46 (54.1)	73 (68.9)	81 (92.0)	

* Shows a significantly higher proportion at the 5% level of significance (based on the chi-square test). Values given in parentheses are percentages.

### 3.1 Logistic regression

Logistic regression analysis, consisting of a binary dependent variable (i.e., high PRA and moderate PRA) and a panel of 12 independent covariates, uncovered 9 predictors of high PRA except for nationality, age, and occupation. Males (versus females) were up to 3 times more likely to have high PRA (OR = 2.95). Married participants had an exposure of high PRA that was almost 2.5 times greater than among those who were single. Those with a history of DM were 4 times more likely to exhibit high PRA, and those with a history of HTN were 3 times more likely to have high PRA. Those with active DM and who were active smokers were up to 5 times more likely to have high PRA. Those with a BOP > 25% had a 3.5 times greater risk of having high PRA. The highest predictive factors of high PRA were > 8 pockets that were ≥ 5 mm (OR = 16.2) and > 8 missing teeth; individuals with these characteristics were up to 5 times more likely to be exposed to high PRA (Table 3).

**Table 3:** Predictive factors of high PRA.

Factors	High PRA (n = 85)	Moderate PRA (n = 108)	OR (95% CI)	P-value
Male	48 (56.5)*	33 (30.6)	2.95 (1.63–5.33)	≤0.001
Saudi national	67 (78.8)	79 (73.8)	1.32 (0.67–2.59)	0.422
Age > 40 years	61 (71.8)*	56 (51.9)	1.00 (0.56–1.76)	0.990
Married	72 (87.8)*	83 (80.6)	2.55 (1.32–4.93)	0.005
Employed	40 (47.1)*	34 (31.5)	0.85 (0.46–1.60)	0.625
History of DM	25 (29.4)*	10 (9.3)	4.08 (1.83–9.09)	≤0.001
HTN	18 (21.2)*	9 (8.3)	2.95 (1.25–6.97)	0.013
Active DM	27 (31.4)*	9 (8.3)	5.12 (2.25–11.6)	≤0.001
Smoking	36 (42.4)*	13 (12.0)	5.37 (2.61–11.1)	≤0.001
BOP > 25%	57 (67.1)*	40 (37.0)	3.46 (1.90–6.29)	≤0.001
> 8 pockets ≥ 5 mm	45 (52.3)*	7 (6.6)	16.2 (6.76–39.0)	≤0.001
> 8 missing teeth	24 (28.2)*	8 (7.5)	4.92 (2.08–11.6)	≤0.001

* Shows a significantly higher proportion at the 5% level of significance.

## 4 Discussion

The evaluation of possible risk factors for disease progression in a specific patient would empower the clinician to establish the extent and frequency of SPT required to maintain the clinical attachment levels attained following non-surgical/surgical periodontal therapy (Trombelli et al., 2020). Hence, this cross-sectional study aimed to evaluate factors associated with a moderate and high risk of PD in Saudi patients.

Various risk assessment tools are employed to determine the potential risk of disease progression in individual patients. We used the PRA (Lang and Tonetti, 2003), which divides individual risk stratification into three categories (low, moderate, and high); 30.5% of the examined patients were at high risk, 38.3% at moderate risk, and 31.2% at low risk. To guarantee the stability of a patient’s periodontal condition, a proper recall interval for SPT should be established after the successful treatment of periodontitis (Trombelli et al., 2015, Tonetti et al., 2015, Chapple et al., 2018). The planning of recall intervals is primarily based on PRA tools after the successful accomplishment of non-surgical/surgical periodontal therapy. SPT is advantageous to maintain periodontally stable dentition and to avoid future tooth loss (Trombelli et al., 2020). Nevertheless, there is a lack of strong evidence regarding the effectiveness, financial impact, and risk/benefits of different recall intervals.

Our outcomes indicate that older adults have a higher risk of PD, as well as a faster rate of disease progression compared to younger individuals. Our findings are in accordance with many earlier studies (Baelum et al., 1988, Genco, 1996) that demonstrated that the prevalence and the severity of periodontal periodontitis rises with age (Rheu et al., 2011, Nazir, 2017, Helmi et al., 2019). The average annual rate of bone loss reported for those aged 70 or above is 0.28 mm, in comparison to 0.07 mm for younger subjects (25 or younger) (Papapanou and Wennstrom, 1989).

Our data agree with similar findings in which male were at greater risk of developing periodontal infection, with a significantly higher risk of disease progression compared to females (Grossi et al., 1995, Mundt et al., 2007). The causes for these gender-related variances are not clear; however, these differences may be associated with the low acquiescence of oral hygiene practices, which is commonly seen among males (Slade and Spencer, 1995, Albandar and Kingman, 1999).

Our results reveal that married Saudi patients are at higher risk of periodontitis compared to those who are single. If one spouse has periodontitis, there is a greater chance that his/her partner will also have periodontitis; hence, married patients are at greater risk of developing periodontitis. Moreover, in one study, male subjects had a poor perception of their periodontitis risks or believed that periodontitis could not be a significant health problem for them (Persson et al., 2004).

In the present study, we observed a stronger association between DM and development, as well as the risk of progression of periodontitis (Genco et al., 2020). PD is the sixth oral manifestation of DM. Patients with poorly controlled or undiagnosed DM have a significantly higher susceptibility to the development and progression of periodontitis compared to non-diabetic patients (Seppala et al., 1993, Stegeman, 2005). In contrast, well-controlled diabetic individuals respond positively to periodontal treatment and can maintain periodontal health (Pucher and Stewart, 2004).

Our results demonstrate a higher risk of PD progression (high PRA) among patients with HTN and ischemic heart disease. Many studies have suggested a correlation between PD and CVD (Sanz et al., 2010, Cho et al., 2021, Tiensripojamarn et al., 2021). This is explained by numerous biological mechanisms, such as how periodontitis might provoke a systemic inflammatory response (Mattila et al., 2002), and the fact that an individual may be predisposed to CVD given the rich reservoir of many anaerobic micro-organisms and the host’s response. In addition, both illnesses share various common risk factors and some obvious similarities in their fundamental pathophysiology (Liccardo et al., 2019, Zhu et al., 2000).

Our results point to a strong correlation between tobacco smoking and PD. Smoking has several destructive influences on the periodontium and exacerbates the rate of PD progression (Özçaka et al., 2011, Bergstrom, 2014). Smoking is known to alter the host’s immune response and his/her ability to challenge the bacterial load associated with PD (Matuliene et al., 2008, Shchipkova et al., 2010).

In this study, a BOP > 25% and pockets ≥ 5 mm were associated with high PRA. The current literature suggests that the severity of periodontitis is a central factor that dictates the interval of maintenance visits (Trombelli et al., 2020). Matuliene et al. (2008) evaluated the effect of different risk factors such as pocket probing depth (PPD) and BOP on mean annual tooth loss during SPT. In that retrospective study, 172 patients were examined after SPT for a period of 3–27 years. A PPD of >5 mm was associated with a higher risk factor of tooth loss compared to a PPD of 3 mm at site and tooth level.

We did not use the sixth factor of the PRA (loss of periodontal support in relation to age) in our analysis due to doubt that the measurements were all standardized, and to exclude any bias that might affect the reported data. Further, we may have underestimated the actual risk for further disease progression; hence, loss of periodontal support in relation to age would be more helpful after periodontal treatment (versus before) (Lang and Tonetti, 2003).

Limitations of our study was the relatively small sample size and that we conducted the study among patients visiting a single dental hospital. Thus, future studies should be performed at multicenter level while considering the use of standardized clinical parameters and standardized examiners.

## 5 Conclusions

Moderate and high-risk periodontitis groups represented more than half of the studied sample. The presence of periodontal pockets ≥ 5 mm, active DM, and increased number of missing teeth were the main factors of high periodontal risk.

## Ethical statement

Ethical approval was granted by Ethics Research Unit of College of Dentistry (#(#2018024), Imam Abdulrahman Bin Faisal University.

## Funding

This research did not receive any specific grant from funding agencies.

## Declaration of Competing Interest

The authors declare that they have no known competing financial interests or personal relationships that could have appeared to influence the work reported in this paper.
